# The Cross-Talk Between Sphingolipids and Insulin-Like Growth Factor Signaling: Significance for Aging and Neurodegeneration

**DOI:** 10.1007/s12035-018-1286-3

**Published:** 2018-08-23

**Authors:** Henryk Jęśko, Adam Stępień, Walter J. Lukiw, Robert P. Strosznajder

**Affiliations:** 1 Department of Cellular Signalling, Mossakowski Medical Research Centre, Polish Academy of Sciences, Warsaw, Pawińskiego 5, 02-106 Poland; 2 Central Clinical Hospital of the Ministry of National Defense, Department of Neurology, Military Institute of Medicine, Warsaw, Szaserów 128, 04-141 Poland; 3 LSU Neuroscience Center and Departments of Neurology and Ophthalmology, Louisiana State University School of Medicine, New Orleans, USA; 4 Laboratory of Preclinical Research and Environmental Agents, Department of Neurosurgery, Mossakowski Medical Research Centre, Polish Academy of Sciences, Warsaw, Pawińskiego 5, 02-106 Poland

**Keywords:** Ceramide, Sphingosine-1-phosphate, Aging, Neurodegeneration, Insulin-like growth factor, Mitochondria

## Abstract

Bioactive sphingolipids: sphingosine, sphingosine-1-phosphate (S1P), ceramide, and ceramide-1-phosphate (C1P) are increasingly implicated in cell survival, proliferation, differentiation, and in multiple aspects of stress response in the nervous system. The opposite roles of closely related sphingolipid species in cell survival/death signaling is reflected in the concept of tightly controlled *sphingolipid rheostat*. Aging has a complex influence on sphingolipid metabolism, disturbing signaling pathways and the properties of lipid membranes. A metabolic *signature* of stress resistance-associated sphingolipids correlates with longevity in humans. Moreover, accumulating evidence suggests extensive links between sphingolipid signaling and the insulin-like growth factor I (IGF-I)-Akt-mTOR pathway (IIS), which is involved in the modulation of aging process and longevity. IIS integrates a wide array of metabolic signals, cross-talks with p53, nuclear factor κB (NF-κB), or reactive oxygen species (ROS) and influences gene expression to shape the cellular metabolic profile and stress resistance. The multiple connections between sphingolipids and IIS signaling suggest possible engagement of these compounds in the aging process itself, which creates a vulnerable background for the majority of neurodegenerative disorders.

## Sphingolipid Biosynthesis and Signaling

Bioactive sphingolipids: ceramide, ceramide-1-phosphate (C1P), and sphingosine-1-phosphate (S1P) play numerous roles in nervous system development and in the acquisition of the mature neuronal phenotype, and as such are key regulators of cell proliferation, differentiation, survival, and the stress response [1, 2]. Their opposite influence on cell survival/death signaling is reflected in the concept of highly regulated *sphingolipid rheostat* and justifies their vast importance in aging and neurodegeneration [3, 4]. Mutations or loss of sphingolipid metabolism enzymes frequently lead to neuronal dysfunction and degeneration or are embryonically lethal [5–7]. Sphingolipids can be secreted into extracellular medium and bind cell surface receptors. They also interact with intracellular signaling pathways [8], bind transmembrane domains of signaling proteins *within* the lipid bilayer [9], or even create membrane pores in mitochondria [10]. Sphingolipids can also modify the operating environment of target proteins through their structural roles as membrane components, potentially facilitating signal amplification and/or the integration of multiple biological signals.

### The Three Pathways of Ceramide Biosynthesis

Ceramide has mostly attracted attention due to its roles not only in cell death and senescence but also in differentiation, maintenance of axonal/synaptic structure, and its links with immunological activation [11, 12]. Ceramide also plays important structural roles in organellar and cellular membranes and their microdomains, including lipid rafts, modulating membrane fluidity, and the biophysical mechanisms of protein anchoring [13, 14]. As a signaling molecule, ceramide is known to bind specific motifs in protein kinases, phosphatases, calcium-binding proteins, DNA repair and heat shock proteins [12, 15, 16]. Ceramide-induced processes such as axonal degeneration/apoptosis comprise both caspase-mediated and caspase-independent pathways involving mitochondrial reactive oxygen species (ROS), p53, Akt, glycogen synthase kinase 3β (GSK-3β, which phosphorylates tau), or the transcription factor activator protein 1 (AP-1) [11, 17–19]. However, some ceramide synthase (CerS) isoforms and ceramide species may have opposite effect on apoptotic and autophagic cell death [20, 21]. Ceramides’ significance for aging and neurodegeneration is also linked to their role in the mitochondrial quality assurance pathways.

**Fig. 1:**
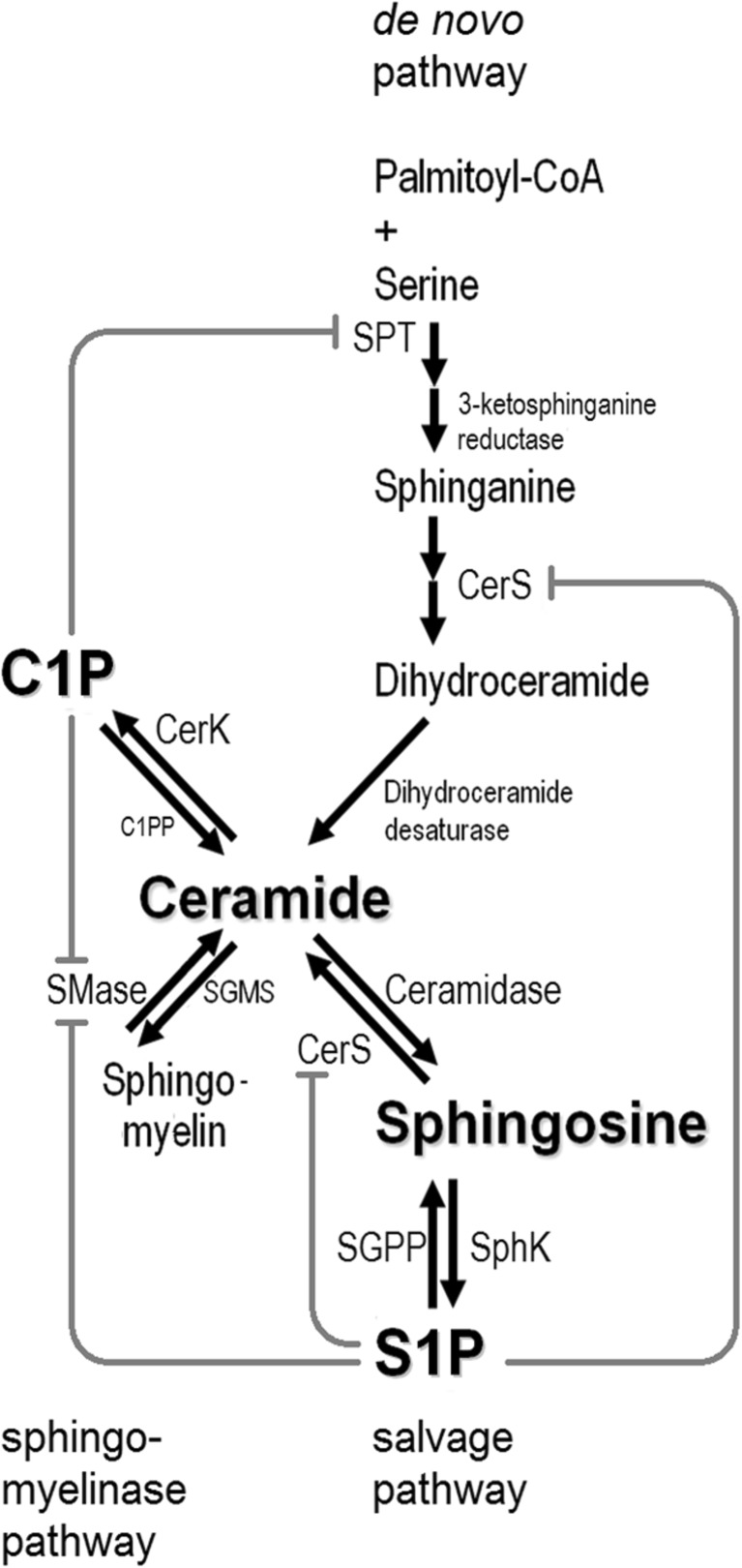
Sphingolipid metabolism and the three pathways of ceramide biosynthesis. The scheme shows only selected reactions and enzymes, plus their feedback regulation by S1P and C1P. C1P, ceramide-1-phosphate; C1PP, C1P phosphatase; CerK, ceramide kinase; CerS, (dihydro)ceramide synthase; S1P, sphingosine-1-phosphate; SGMS, sphingomyelin synthase; SGPP, S1P phosphatase; SMase, sphingomyelinase; SPT, serine palmitoyltrasnferase

Three main pathways of ceramide generation have been described: de novo biosynthesis from serine and palmitoyl-coenzyme A, the *sphingomyelinase pathway*, and the *salvage pathway* that re-creates ceramide from sphingosine (Fig. 1):

- Serine palmitoyltransferase (SPT) catalyzes the first, rate-limiting step of sphingolipid biosynthesis. SPT product is then converted into sphinganine (dihydrosphingosine) which is used by CerS to generate dihydroceramide [22], which recently emerges as a signaling molecule on its own [23, 24]. Dihydroceramide is then converted to ceramide by dihydroceramide desaturase.
- Ceramides can give rise to sphingomyelin [25] produced by sphingomyelin synthase (SGMS or SMS). The opposite reaction catalyzed by sphingomyelinases (SMases or SMPDs), termed the *sphingomyelinase pathway* is a major ceramide source [26].
- Ceramide can be further converted by ceramidases into sphingosine [27] which modulates the cell death machinery and nitric oxide (NO) signaling [28, 29]. Thanks to their relatively broad substrate specificity, ceramide synthases (there are CerS1 to 6, also named longevity assurance gene homologs Lass1 to 6) can re-synthesize ceramide from sphingosine (the *salvage pathway*). The presence of six tightly regulated and interdependent ceramide synthases plus their splice variants assures the necessary variation in synthesized ceramides [30]. CerSs are known to undergo phosphorylation, acetylation, *N*-glycosylation, and ubiquitination, implying tight (but still poorly understood) regulation [30, 31].

### Sphingosine-1-Phosphate and Ceramide-1-Phosphate

Both sphingosine and ceramide can be phosphorylated into their respective 1-phosphates (S1P and C1P). For years, the roles of bioactive sphingolipids have been interpreted using the *sphingolipid rheostat* model which implies survival-promoting activities of sphingolipid phosphates in contrast with the pro-apoptotic ceramide. Although the model still seems to correctly describe the prevailing significance of each compound class, the roles are no longer clear cut.

The pro-survival activity of S1P highlights its role in brain physiology and the potential significance as therapeutic target in neurodegenerative disorders [32–34]. S1P mediates the actions of numerous anti-apoptotic compounds such as nerve growth factor or glial-derived neurotrophic factor [4]. Largely through phosphoinositide 3-kinase (PI3K)-Akt, the sphingosine kinase (SphK) signaling targets pro-apoptotic proteins Bad (Bcl-2-associated agonist of cell death) and GSK-3β [17] and nuclear transcription factors including known regulators of apoptosis. The latter include forkhead box, sub-group O transcription factors (FOXO) [35], NF-κB [8, 36], and AP-1 which is also engaged in the network of mutual co-regulation between sphingolipid-related genes [37–39]. However, prolonged accumulation of S1P (produced by SphK2) can cause endoplasmic reticulum stress and cell death [4, 40]. Some of S1P’s mediators, such as AP-1 [41], extracellular signal-regulated kinases (ERK) [42, 43], or NF-κB [44, 45] can also lead to various neurological outcomes [40–45].

S1P production by SphKs undergoes extensive regulation by numerous inputs including growth factors, inflammatory cytokines, or calcium ions [46, 47]. S1P is dephosphorylated back to sphingosine by phosphatases SGPP1 and SGPP2. S1P can also be irreversibly hydrolyzed by the SGPL lyase [48] into hexadecenal (which has its own signaling functions [49]) and ethanolamine phosphate.

**Fig. 2:**
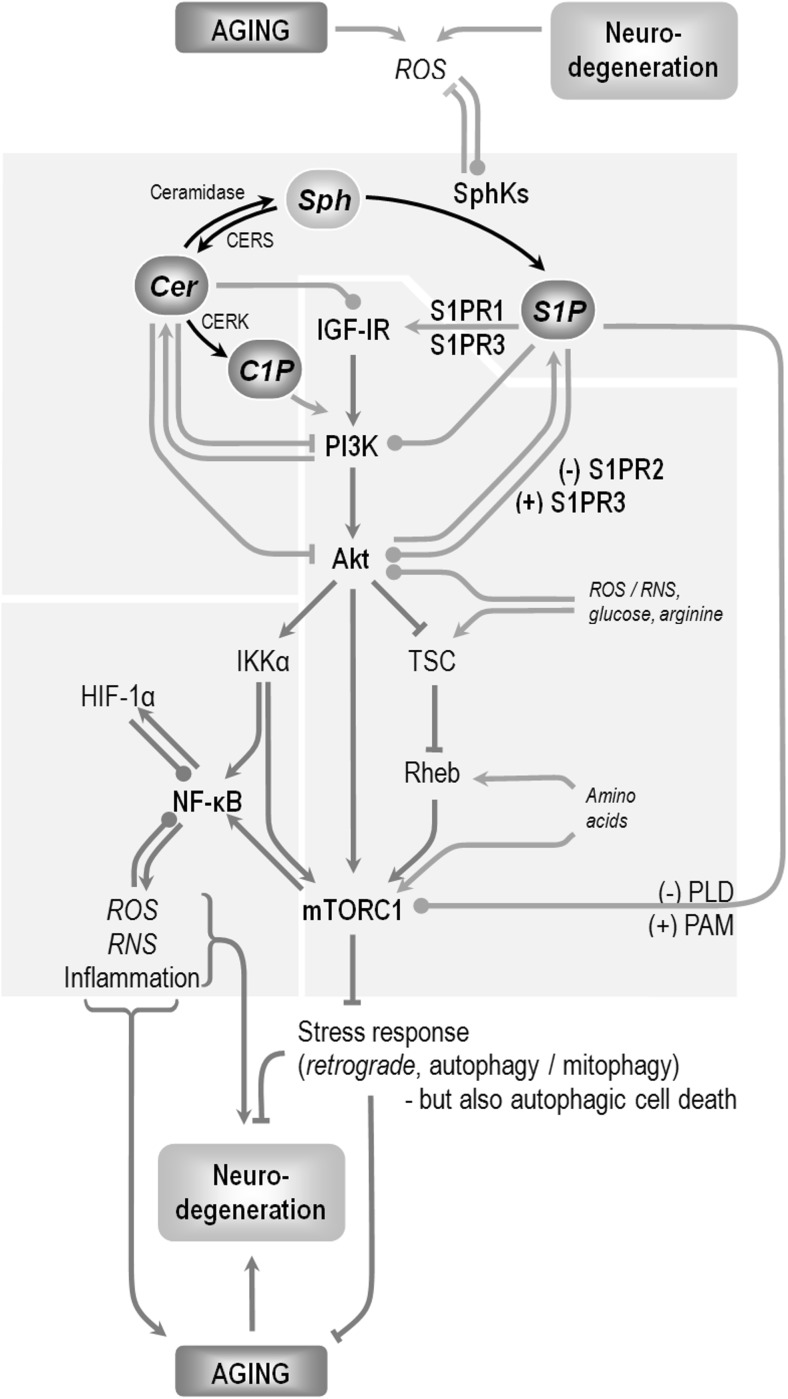
Modulation of the PI3K-Akt-mTOR signaling by bioactive sphingolipids. Bi-directional interactions with cellular stress, aging, and neurodegeneration. Selected enzymes of sphingolipid metabolism with known significance for the modification of the aging process are shown. Akt, protein kinase B; C1P, ceramide-1-phosphate; Cer, ceramide; CerK, ceramide kinase; CerS (LASS), ceramide synthase; HIF-1α, hypoxia-inducible factor-1α; IGF-IR, insulin-like growth factor receptor; IKK, inhibitor of NF-κB kinase; mTORC1, mammalian (or: mechanistic) target of rapamycin complex 1; NF-κB, nuclear factor κB; PAM, protein associated with Myc; PI3K, class I phosphoinositide 3-kinase; PLD, phospholipase D; Rheb, Ras homolog enriched in the brain; RNS, reactive nitrogen species; ROS, reactive oxygen species; S1P, sphingosine-1-phosphate; S1PR, cell surface G protein-binding S1P receptors; Sph, sphingosine; SphK, sphingosine kinase(s); TSC, tuberous sclerosis protein.  symbols denote varied/ambiguous influence (e.g., depending on the cell model used, the presence of RNS has been found to either activate or inhibit IKKβ). The scheme skips additional interactions, such as the links between ceramide itself and stress, the inhibitory phosphorylation of TSC1 by IKKβ, or feedback influences of mTOR on PI3K-Akt. Sphingolipids can modulate mTOR in multiple ways, potentially allowing cell type/subcellular compartment-specific functioning of the S1P-mTOR branch. mTORC1 activation occurs on the surface of various organellar membranes, and details of the pathway may be different depending on the cell region. According to [3, 52], modified

S1P can both play the role of an intracellular second messenger, or act on multiple cell types through surface S1P receptors in auto-/paracrine fashion. S1P can be transported to more distant targets in the cerebrospinal fluid or in the bloodstream [1, 11, 12, 50]. The cell surface, low-nanomolar affinity S1P receptors of the Edg family (termed S1P1 to 5) bind G_q_, G_i_, G_12/13_, and Rho proteins which relay signals to PI3K, protein kinase C (PKC), phospholipases, or cyclic adenosine monophosphate (cAMP) [51] (Fig. 2). S1PRs influence neuronal viability, excitability, and neurite extension/retraction [53]. S1PRs also modulate the interactions between neurons and microglia and possibly decide about the outcome (restorative vs. neurotoxic) of astroglial immune activation [53, 54]. The nervous system is enriched in S1PR proteins, especially S1PR1 (whose expression changes with age), S1PR3, and S1PR5. S1PR2 undergoes low-level, gender-specific brain expression [55]. Neurons, astrocytes, and microglia express S1PR1–3 and S1PR5, while oligodendrocytes and their precursors possess S1P1, S1P3, and S1P5 [53, 56]. Cell surface receptor-mediated S1P signaling includes feedback effects such as reduction of SphK1 expression in response to S1PR2 activation or ligand-induced receptor internalization (this phenomenon is exploited in the therapy of relapsing remitting multiple sclerosis that employs fingolimod, a S1P receptor modulator [53]), [57, 58].

The second messenger function requires S1P generation to occur in various intracellular compartments including nucleus. Although nuclear pores should allow exchange of sphingolipids with the cytoplasm, their nuclear pools show large degree of autonomy, and sphingolipid metabolism enzymes exist in the nucleus (including ceramide and sphingosine kinases, sphingomyelin synthase, and sphingomyelinase) [51]. SPHK2 has been found to participate in repressor complexes with histone deacetylases (HDAC1 and 2), while S1P can bind both HDAC proteins and inhibit their deacetylase activity [59]. These varied mechanisms of nuclear signaling appear to be important for inflammaging and neurodegenerative conditions (Alzheimer’s disease (AD)), along with the above mentioned sphingolipid-mediated modulation of transcription factors [49, 60, 61].

C1P stimulates cellular proliferation and survival and antagonizes ceramide (Fig. 2) [62–67]. However, C1P can be cytotoxic at high concentrations [68], and it can stimulate ROS-based signaling and lead to induction of the NO synthase iNOS [69, 70]. Surprisingly, at least in some cases, Akt- and NF-κB-dependent iNOS stimulation might actually mediate the pro-survival effects of C1P [70]. C1P employs several mechanisms to exert its influence on downstream mediators. While it can bind its intracellular target enzymes directly [71, 72], it can also cross the plasma membrane [68, 73] and Granado et al. suggested the existence of a specific, low affinity plasmalemmal C1P receptor that signals through G_i_ protein to the known mediators of C1P activities: PI3K/Akt, NF-κB, and mitogen-activated protein kinase kinase (MEK)/ERK [74].

## Bioactive Sphingolipids in Aging

Numerous alterations in sphingolipid metabolism are observed during human and rodent aging (Table 1). A characteristic set of lipids possibly linked to stress resistance has been found to correlate with longevity [80]. The known association of sphingolipids and IIS with immune signaling also hints at their potential significance for inflamm-aging, which is important for the pathological, real-life trajectories of homoeostasis deterioration in old age [93, 94]. In humans, the hippocampal sphingolipid balance tends to change with age towards ceramide and sphingosine; this likely contributes to the worsening of the conditions for neuronal survival [83].

**Table 1:** Changes of sphingolipid metabolism/signaling in aging and AD

Aging	AD
↑ membrane neutral SMase activity. ↓ cytosolic neutral SMase activity [75]	↑ human AD brain mRNA levels: *CERS1*, *CERS2*, *SGPL1*, *SPTLC2*, acid SMase [76, 77], although CerS2 activity is reduced [78]. ↓ human AD brain mRNAs: *ASAH1*, *CERK*, and *CERS6* [76]. ↓ human SphK1 and SphK2 activity in the hippocampus [79]; specific SphK2 activity increased in AD frontal cortex [78]
A “signature’”set of lipids associate with human longevity [80]	Human brain sphingomyelin and hydroxysphingomyelin species correlate with the future AD onset in asymptomatic/healthy subjects, and with progression at the pre-symptomatic/prodromal stages [81]. Serum levels of selected sphingomyelins correlate with progression from MCI to AD [82]
↓ S1P/sphingosine ratio in human aging hippocampus (only in females) [83]	↓ S1P levels in human AD brain hippocampus and inferior temporal cortex; hippocampal changes correlate with Braak stage of neuropathology [79]
↑ human hippocampal ceramide [83]. ↑ ceramide/sphingomyelin ratio in the rat serum, liver, heart, and skeletal muscle [84]. ↑ various human ceramide species [85]. ↑ ceramide/sphingomyelin ratio in the rat serum, liver, heart, and skeletal muscle [84]. Contradictory changes of various ceramides in centenarians [80]	↑ human brain ceramide levels—including the earliest clinically recognizable AD stage (MCI) [77, 86–88]
↑ human hippocampal sphingomyelin level [83]. ↑ sphingomyelins in the mouse hippocampus [89] and rat amygdala [90]. ↑ human blood plasma sphingomyelins [91]	Incoherent results on sphingomyelin changes in AD patient serum [92]

The table includes only observations in naturally aging humans and rodents and from human AD cases

*ASAH1*, an acid ceramidase; *C1P*, ceramide-1-phosphate; *C1PP*, C1P phosphatase; *CerK*, ceramide kinase; *CerS*, (dihydro)ceramide synthase; *MCI*, mild cognitive impairment; *S1P*, sphingosine-1-phosphate; *SGMS*, sphingomyelin synthase; *SGPL*, S1P lyase; *SGPP*, S1P phosphatase; *SMase*, sphingomyelinase; *SPT*, serine palmitoyltrasnferase; *SPTLC*, SPT long-chain base subunit 1

The knowledge of the mechanisms of sphingolipid involvement in human aging/longevity is highly incomplete. Most work has been done on yeast and nematode models, and results draw attention to the extensive cross-talk between sphingolipids and IIS.

### Ceramides in the Stress Response and Cellular Senescence

Evidence from yeast studies points to the links between ceramide metabolism and aging [95, 96]. A CerS subunit gene has been identified as *longevity-assurance gene 1* (*LAG1*) in the yeast replicative senescence model [25]. The effects of its manipulation on the lifespan are complex, while its mild overexpression increases the replicative potential, higher levels lead to its reduction [97]. However, in *Drosophila* ablation of an alkaline ceramidase can improve lifespan and oxidative stress resistance [98]. Results obtained in *Caenorhabditis elegans* suggest that age-related changes in relative concentrations of ceramide subspecies are absent in the long-living calorie-restricted adults or *dauer* larvae, pointing to the potential significance of fine-tuning of ceramide metabolism for the aging process [99]. Inhibition or knockdown of acid sphingomyelinase, serine palmitoyltransferase, or glucosylceramide synthase in *C. elegans* lead to longer lifespan; the effects are partially mediated *via* IIS signaling [99, 100].

The known links between ceramides and stress, which is one of the driving forces of aging [101–104], are extensive also in mammalian tissue.

- Ceramides seem to respond to the inefficiencies in the ROS control. Ceramide content is elevated early in the Cu/Zn SOD (superoxide dismutase) mouse mutants and in human amyotrophic lateral sclerosis patients with this mutation [105]. It is suggested that physiological glutathione (GSH) levels inhibit neutral sphingomyelinase (nSMase), and the enzyme’s activity only rises when glutathione is depleted by, e.g., oxidative or alkylating agents or cell senescence [106].
- Ceramide levels can also be enhanced by the oxidative stress sensor p53, a protein engaged in the regulation of aging/senescence [107].
- Ceramide and the enzymes of its metabolism are linked bi-directionally with AP-1, a redox-sensitive transcription factor engaged in cellular senescence, responses to oxidative stress and DNA damage [108]. Cytoprotective effects of serum growth factors include activation of the neutral ceramidase gene via AP-1 [39]. However, also *CERS4* and *5* genes are activated by AP-1. Thus, the complex regulation of AP-1 may enable it to stimulate or suppress ceramide levels [38]. In turn, ceramide inhibits AP-1, creating a feedback loop ensuring tight control over its own concentration [19, 109].

### Sphingosine-1-Phosphate, Stress Signaling, and Senescence

Despite extensive links between S1P and proliferation control, the significance of S1P for the modulation of cellular senescence is poorly characterized. Inhibition of SphK1 leads to p53- and p21-dependent senescence in a human cell line [110]. SphK1 reacts to oxidative stress in a apparently bimodal fashion. While moderate stress activates it, high ROS production can lead to its inhibition and/or degradation. This phenomenon probably reflects a switch that occurs in excessively damaged cells, which directs the resources away from building stress resistance, and instead activates apoptosis [3]. This switch reflects the behavior of p53, and p53 is indeed upstream of SphK1 (p53 can activate SphK1 degradation by cysteine proteases) [111, 112]. In turn, SphK1 seems to suppress ROS production [113], and this phenomenon significantly contributes to the known protective effects of ischemic preconditioning, as shown in the heart [114]. The dualistic reaction of SphK1 to oxidative stress has led to a proposal that it could be engaged in the longevity control, probably via its links with ROS and their sensor hypoxia-inducible factor (HIF-1) [3].

The ability of sphingosine kinases to modulate ceramide metabolism (Fig. 1) may have additional impact upon stress signaling and resistance. SphK1 can influence ceramide synthesis on the *de novo* and salvage pathways by changing CerS1 intracellular localization and probably SPT and CerS activities [115–117]. In turn, SphK2 overexpression can lead to increased ceramide synthesis [117].

S1P produced by SphK2 binds human telomerase catalytic subunit in human and mouse cells, preventing its ubiquitin-dependent degradation. Disruption of telomerase–S1P binding leads to telomere erosion and acquisition of the senescent phenotype [118]. In turn, up-regulated S1PR2 expression occurs in senescent cells of various types, and S1PR2 activity has been demonstrated to support cellular senescence [57, 119, 120].

The expression and activity of the transcription factor AP-1 is also dependent on S1PR1/S1PR3 signaling [121], suggesting further roles for S1P in cellular senescence. A positive regulatory loop appears to exist between SphK1 and AP-1, as the *SPHK1* gene contains AP-1 binding elements, and its expression is dependent on c-Fos and c-Jun [37].

### Bioactive Sphingolipids and the Insulin-Like Signaling Pathway of Aging Modulation

Perhaps the most promising (though still incompletely characterized) mechanism of metabolic and stress control by sphingolipids is mediated by the versatile IIS pathway. IIS, a highly inter-connected metabolic regulatory system, is implicated in stress resistance/aging modulation throughout the spectrum of organisms from nematodes to vertebrates [122, 123]. Interestingly, many roles of S1P appear to be largely analogous to those of IIS, including not only the well-documented cell survival/death signaling but also the engagement in organism’s energy homoeostasis [124].

IGF-I receptor (IGF-IR) signaling is tightly associated with lipid rafts, which might sensitize it to the structural influence of sphingolipids on cell membrane microdomain properties [125]. Accumulating evidence also suggests links between signaling activities of sphingolipids and the wide spectrum of IIS activities (Fig. 2). Increased SphK1 expression and S1PR1/S1PR3 signaling are engaged in the IGF-IR activation [126]. C2 ceramide alters the expression of several IIS genes in a tissue-specific manner, including reduced IGF-IR and insulin receptor substrate IRS-1 or elevated IRS-2 and IGF-binding protein 1 (IGFBP1) in liver cells [127, 128]. IGFBPs are carrier proteins that not only regulate IGF-I bioavailability but can also have IGF-independent modulatory influence on cell survival [129]. nSMases have been shown to modulate the expression of IGFBP1 via FOXO1 [128, 130]. In the nematode-aging model, a number of ceramide-synthesizing enzymes signal largely through IIS, limiting the lifespan as mentioned previously [99, 100]. Worm *sphk-1* mutants live shorter and are more susceptible to heat stress [131].

Phosphatidylinositol 3-kinase (PI3K) receives signals from plasma membrane receptors that bind growth factors (IGF-IR), hormones (insulin), and from chemokines [132].

- S1P synthesis by both SphKs has been found to be activated by IGF-I [133, 134], at least partially *via* its signaling target Akt [135]. S1P in turn influences the activities of PI3K and Akt (Fig. 2); this might add an important modulatory loop to the IGF-IR-PI3K signaling. The influence of S1P on PI3K depends on several factors, including PI3K and SphK isoform and its intracellular localization [136]. As mentioned, while pro-apoptotic in some circumstances, S1P produced by SphK2 might also promote cell survival through PI3K-Akt [137]. The interaction of S1P with PI3K-Akt appears to engage nearly the whole repertoire of the sphingolipid’s signaling mechanisms. S1PR1 and S1PR3 can activate PI3K and Akt via G_i_. S1PR2 ligation may lead to Akt inhibition (probably through G_12/13_ and PTEN), thus the outcome of S1P signaling to IIS via the cell surface is dependent on the cell type [138–142]. However, S1PR2 response includes feedback reduction of the receptor expression [143], probably explaining why S1PR2 can in some situations functionally augment the PI3K-Akt signaling [143, 144]. Kim et al. published data suggesting S1PR-independent, second messenger-like negative effects of S1P on Akt [145].
- The typically negative influence of ceramide on the IIS-dependent pro-survival signaling [146] includes dephosphorylation of Akt by ceramide-activated protein phosphatase (CAPP) and protein phosphatase 2A (PP2A), followed by modification of Akt subcellular distribution [147, 148]. Inhibition of Akt by C6 ceramide has been shown to involve PKCζ [149]. In turn, PI3K has been shown to block ceramide synthesis [150]. As part of its anti-apoptotic activity, Akt can also modulate ceramide transport between endoplasmic reticulum and Golgi apparatus, additionally influencing ceramide bioavailability for the synthesis of complex sphingolipids [151].
- C1P stimulates the activity of PI3K and Akt, leading to cell proliferation and reduced apoptosis [152, 153].

Although highly fragmented and sometimes incoherent, current data suggest extensive engagement of sphingolipid signaling in the modulation of IIS at several levels. Evidence is accumulating that the influence of sphingolipid signaling, mostly observed at relatively upstream levels of the IIS (IGF-IR, PI3K, Akt), can indeed lead to meaningful modulation of known aging-related targets of the pathway.

#### The Divergent Roles of Insulin-Like Growth Factor Signaling (IGF-IR, PI3K, Akt) in Organism Longevity and in Brain Aging: the Potential Role of Sphingolipid Signaling

The significance of IIS is vast for both physiological aging and the age-related neurodegenerative disorders. Despite of the involvement of the brain IGF-I signaling in the modulation of whole-organism longevity, the influence of IIS on the condition of the brain itself appears to be very different from its role in the periphery*,* and results are inconsistent [154, 155]. In-depth elucidation of the trophic role of IIS and its dysfunction in brain aging is ongoing [156–158], bridging the numerous gaps in our current understanding of the molecular events leading to the creation of the disease-promoting environment of the aged CNS.

In turn, the groundbreaking discoveries of last decades point to IIS as the crucial pathway that re-directs vital resources towards short-term needs such as energy metabolism, macromolecule synthesis, or survival of individual cells at the expense of the long-term organism maintenance/longevity. Multiple stress stimuli (caloric restriction (CR), starvation, oxidative damage) neutralize the IIS-dependent inhibition of antioxidant defenses (Fig. 2). In nonvertebrates, inactivation of the IIS pathway leads to long-living larval (constitutive *dauer*) or adult forms, typically displaying high resistance to broad range of stress conditions [159, 160]. The role of IIS in vertebrate longevity appears to follow a relatively similar scheme [161, 162]. Like in lower organisms [163, 164], the longevity effect of IIS inhibition in rodents was dependent on signaling events taking place in neurons [165, 166]. Human data seems to support the role of IIS in lifespan determination, as polymorphisms in IIS genes associate with longevity [167], and centenarians show over-representation of gene variants associated with high circulating IGF-I but reduced IGF-IR activity [168]. However, the matter is still not settled [169], and more research is necessary to characterize in depth the boundary between insulin-like signaling and its molecular targets in lifespan determination.

#### The Significance of Sphingolipid-Dependent Modulation of Longevity-Associated IIS/PI3K/Akt Signaling Mediators and Targets

Crucial mediators of Akt signaling (Fig. 2) include mTORC1, a protein complex centered around the serine-threonine kinase mechanistic/mammalian target of rapamycin (mTOR). mTORC1 is activated through several branches of the pathway: through phosphorylation of an mTORC1 subunit, through a cascade of inhibitory signals via tuberous sclerosis protein (TSC) and Ras homolog enriched in brain (Rheb), or through IκB kinase α (IKKα) [170]. These mTORC1-regulating pathways integrate growth factor signals with a vast spectrum of additional factors that reflect cellular metabolic status: oxidative and nitrosative stress, energy/glucose/oxygen levels (sensed, e.g., via AMPK-5′ adenosine monophosphate-activated protein kinase, and relayed to Akt and TSC [171–176]), amino acid availability (arginine through TSC [177], and multiple amino acids via indirect signals converging on Rheb and mTORC1 [178]). They also allow cross-talk with S1P/C1P/ceramide signaling. Sphingolipids can change mTORC1 activity via their influence on PI3K and Akt, but PI3K-/Akt-independent pathways have also been described (Fig. 2):

- S1P activates mTOR through protein associated with myc (PAM), an E3 ubiquitin ligase [179];
- A phospholipase D-mediated mechanism has been reported where S1P might block mTOR-dependent signaling to S6K and 4E-BP1, leading to enhanced autophagy [180].

mTORC1 and its signaling targets are viewed as a major driving force of numerous cellular processes that contribute to aging, including oxidative catabolism, protein and lipid synthesis, and disturbed free radical defenses [181–183]. mTORC1 is engaged in age-related deregulation of proteostasis, nutrient-dependent signaling, mitochondrial metabolism, and in the acquisition of senescent phenotype (including the pro-inflammatory *senescence-associated secretory phenotype* (SASP)) [184, 185]. However, it is worth noting that its positive effects on the respiratory chain [186] can be accompanied by enhanced expression of SOD, catalase, and glutathione peroxidase (GPx) [187].

Changes in mTOR signaling mediate multiple effects of caloric restriction [185]. mTORC1 inhibitor rapamycin is an extremely robust pharmacological treatment that extends lifespan in multiple model organisms, including mammals, even if administered relatively late [185]. Mutation analysis and microRNA research confirm the role of mTOR [188, 189]. The outstanding universality of the lifespan effects of rapamycin have led to suggestions about potential human intervention candidate [185].

**Fig. 3:**
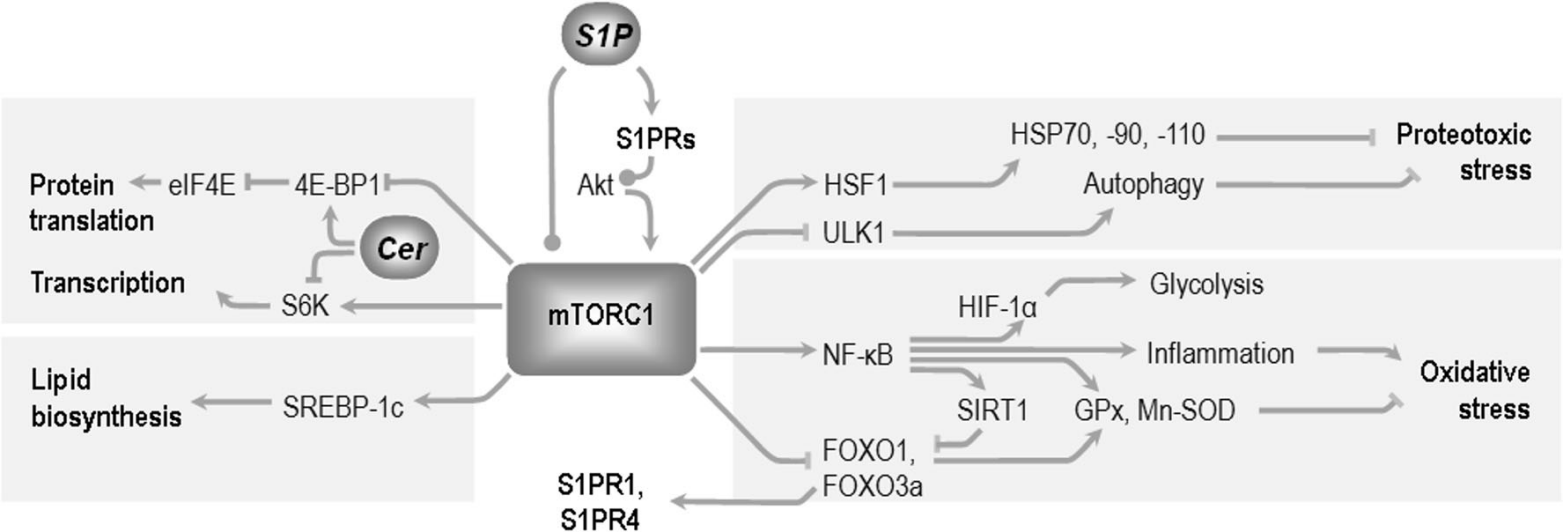
Selected mediators of mTORC1 signaling in the modulation of cellular metabolism and organism lifespan. Fragmented evidence shows that sphingolipids can lead to opposite effects on mTORC1 activity, depending on the mediators engaged, or experimental model used (see text). The mTORC1 complex influences protein quality assurance mechanisms through heat shock transcription factor 1 (HSF1) and the Unc-51-like kinase (ULK1) [190]. mTORC1 stimulates transcription (via ribosomal S6 kinase (S6K)), and translation (through blocking of 4E binding protein 4E-BP1, which itself is an inhibitor of eukaryotic translation initiation factor 4E-eIF4E). 4E-BP1 can react positively to the presence of ceramide, although the mechanism has not been fully elucidated. Sterol regulatory element-binding protein (SREBP-1c) mediates the effect of mTOR on lipid biosynthesis [191]. The extremely pleiotropic functions of NF-κB include regulation of the glycolysis-controlling hypoxia inducible factor 1α (HIF1α), sirtuin (SIRT1), and forkhead box O1 transcription factors (FOXOs, which can send feedback signal to S1P receptors), and antioxidative enzymes glutathione peroxidase (GPx) and manganese superoxide dismutase (Mn-SOD). According to [192], modified

The best characterized mediators of mTORC1-dependent actions include S6K1 and S6K2 (ribosomal protein S6 kinases), 4E-BPs (eukaryotic translation initiation factor 4E-binding proteins), and FOXO transcription factors, but also NF-κB and its interaction partners increasingly seem to play important roles (Fig. 3):

- S6K1 and S6K2 are mTORC1-activated stimulators of protein synthesis. Disruption of S6K1 extends lifespan in mice and recapitulates metabolic aspects of CR (including altered gene expression patterns, insulin sensitivity, and glucose tolerance), suggesting the kinase as a crucial mediator of the robust life-prolonging intervention [193, 194]. Partial inhibition of sphingolipid biosynthesis (pharmacological or genetic reduction of SPT activity) increases yeast cell chronological lifespan through Sch9, an ortholog of S6K [25, 195]. The topic has not been characterized directly in mammals, but experimental data suggest links between sphingolipids and S6K-dependent modulation of aging. Ceramide leads to S6K inhibition [196]; moreover, altered proportions of ceramide species and the resulting disruption of Akt to S6K1 signaling has been suggested to underlie an important aspect of muscle aging—the loss of adaptability to physical effort [197]. The influence of S1P or FTY720/fingolimod on PI3K (positive or negative, depending on the cell type and its S1PRs subset) has been shown to translate respectively into activation or inhibition of S6K [179, 198, 199]. Fingolimod also increases protein levels of mTOR and S6K, and this effect was probably responsible for the reduction of autophagic neuron death [198]. As a feedback mechanism, S6K can block PI3K activation by the insulin receptor. It may sometimes lead to unexpected results such as the presence of activated S6K despite inhibition of the upstream Akt signaling by ceramide [200].
- Phosphorylation of 4E-BP1 and 4E-BP2 by mTORC1 removes their inhibitory influence on the translation regulator eIF4E. Ceramide has been shown to activate 4E-BP1, although not always via Akt/mTOR [201]. 4E-BP participates in a wide spectrum of stress-response mechanisms [202–206] and mediates the effects of diet (CR/reduced amino acid supply), temperature, and probably IIS manipulation, on nonvertebrate longevity [202, 207, 208]. Its activation protects mammalian tissues against metabolic disturbances associated with age [209] while loss of 4E-BP regulation contributes to the mentioned age-related disruption of muscle adaptation [210].
- FOXO transcription factors belong to central modulators of IIS/mTOR-dependent stress resistance/lifespan in organisms ranging from nematodes to humans [101, 211, 212]. FOXOs extensively cross-talk with sirtuins (SIRT-1 to 7; homologs of yeast silent information regulator 2) that sense the cellular metabolic status and stress conditions, and orchestrate stress response/macromolecular repair, influencing the course of aging, neuronal plasticity/learning and memory, and neurodegenerative diseases including AD [101, 211]. FOXO1 and FOXO3a typically undergo Akt- or mTORC1-mediated inhibition [213], which neutralizes their numerous homeostatic activities (FOXOs stimulate the expression of catalase, Mn-SOD, GPX, or peroxiredoxin III) [214–217]. Limited evidence suggests that FOXOs might take part in the effects exerted by sphingolipids *via* IIS. Activation of PI3K and Akt by S1P has been shown to actually trigger the expected downstream events such as inhibitory phosphorylation of FOXO3a or up-regulation of B cell lymphoma 2 (Bcl-2) and B cell lymphoma-extra large (Bcl_xL_), leading to impact on the cell survival [218–220]. Interestingly, FOXO1 exerts feedback regulation upon the expression of S1P receptors (S1PR1 and 4) [221].
- NF-κB is an immune modulator that often contributes to neuronal damage, although the spectrum of its known roles is much wider: sensing oxygen levels, ROS, and RNS (reactive oxygen and nitrogen species) [222–224] (Fig. 2), stimulation of free radical defense, but also of prooxidative enzymes and cell death [225–227]. NF-κB cross-talks with IIS (Fig. 2) and is linked with aging modulation, with cellular senescence, and SASP [225, 227–229]. Moreover, disturbances of the NF-κB target HIF-1α may be responsible for the age-dependent, mitochondria-linked deregulation of energetic metabolism in mammals [230, 231]. Finally, links of NF-κB and the *retrograde response* (see below) deserve further attention in the context of aging mechanisms [232].

S1P activates NF-κB through Akt signaling to either IKKα or mTORC1, leading to modulation of NF-κB target genes and responses [233–235]. However, the significance of this interaction is unclear due to the NF-κB’s above-mentioned role in immune stimulation and cell death—also in the brain [236]. The importance of fine-tuning of various aspects of NF-κB response to S1P signaling is further stressed by the fact that S1P and the S1P receptor modulator fingolimod sometimes paradoxically exert opposite influence on some NF-κB targets such as NO production in astrocytes [236]. Moreover, while some S1P receptors (including S1PR2) activate NF-κB, S1PR5 actually inhibits it, suggesting that the cell type and its relative expression levels of various S1PR isoforms can *switch* the outcome of S1P signaling [235, 237].

The poorly characterized C1P receptor allows also C1P to stimulate DNA binding by NF-κB via PI3K and Akt [152, 153].

Interestingly, DNA binding by NF-κB is also increased in response to a cell-permeable short-chain analog of ceramide, though this response did not lead to increase in the measured gene activities [238]. The significance of high levels of endogenous, C16:0 and C20:0 ceramides for NF-κB has been confirmed by Rivas et al., who found elevated expression of the transcription factor in old muscle, where it probably contributed to the age-related attenuation of the tissue adaptability to exercise [197]. NF-κB exerts feedback responses on various levels of sphingolipid signaling, mainly through up-regulation of SPT and acid sphingomyelinase, but also S1P phosphatase [239–241].

#### The Retrograde Response as an Example of Sphingolipid Role in Aging/Senescence

The significance of the cross-talk between sphingolipids and the IIS-dependent modulation of stress defense has also been analyzed in the context of the retrograde response that signals, e.g., the presence of defective mitochondria, a crucial element of aging, to the nucleus [242]. TORC1 senses the cellular nutrient status, and when glutamate is lacking, reduced TORC1 activity leads to de-repression of the retrograde response, which interacts with the regulatory pathways of mitophagy. Mitophagy is a subset of autophagic organelle degradation, and its proper regulation ensures that defective mitochondria are eliminated leaving the best-preserved organelles for replication. The retrograde response is known to be involved in yeast cell lifespan extension. Its crucial significance for the long-term cellular maintenance has led to the suggestion that the longevity-related function of yeast *LAG1* and its worm orthologs *hyl-1* and *hyl-2* in fact stemmed from their significance in the integration of these mitochondrial quality signaling pathways [243]. The link between the detection of defects by the retrograde response and the execution of mitophagy involves sphingolipid signaling (*LAG* ceramide synthases, ceramidases), TOR, and a TORC1-interacting ortholog of mammalian S6K [243]. Mammalian orthologs of the retrograde response pathway proteins also include NF-κB. The mechanism appears to be related to elements of the mammalian *unfolded protein response* and *endoplasmic reticulum stress* [243, 244]. The importance of degradation of defective mitochondria for neuronal cell maintenance makes it a promising aging research target, although there is a clear difference in the observed effects between rodents and humans [245]. The wide-ranging longevity effects of mild mitochondrial uncoupling/coenzyme Q synthesis manipulation via reduced *MCLK1* (5-demethoxyubiquinone hydroxylase) gene expression suggest the existence of *retrograde-*type signaling in vertebrates [244]. However, the identity of mammalian proteins that signal mitochondrial damage to the nucleus is still not well understood [246].

#### Sphingolipids and Mitochondrial Number/Quality Control

The role of mitochondria in cell death signaling by ceramide has been reviewed extensively [247, 248]. However, evidence is accumulating on ceramide roles in a whole spectrum of regulatory events that affect their function. In mammalian oocytes disturbed intracellular localization of ceramide (due to loss of ceramide transport protein expression) seems to contribute to loss of mitochondrial function with oocyte age, which may be an important example of links between sphingolipid metabolism and long-term cellular homoeostasis [249].

**Fig. 4:**
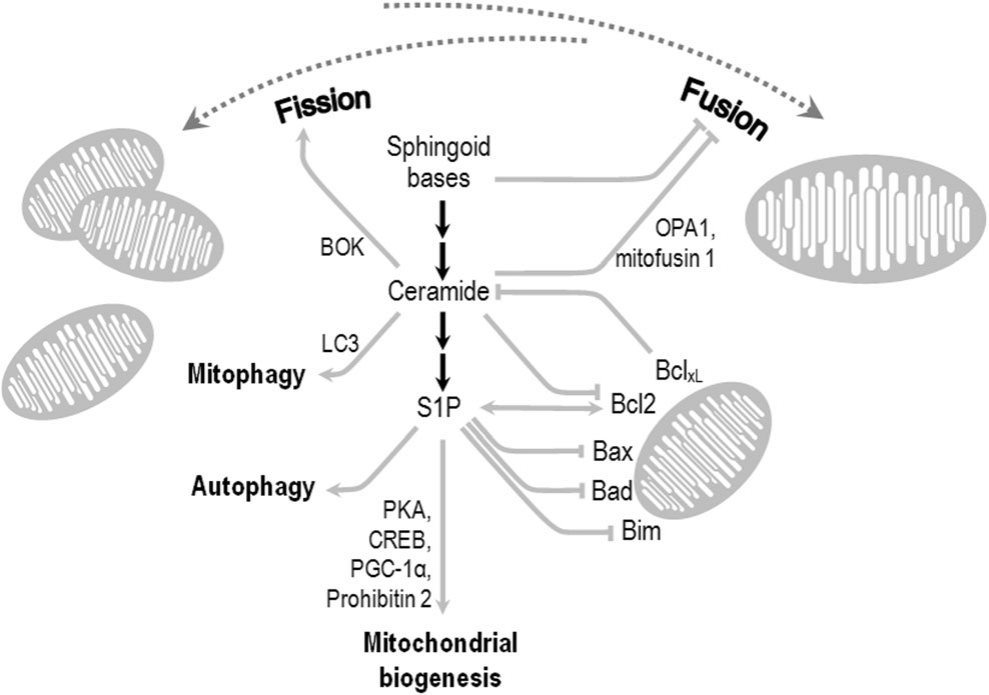
Bioactive sphingolipids and their roles in mitochondrial fusion, fission, autophagy/mitophagy, and apoptotic signaling. Ceramide is capable of activating mitophagy (via microtubule-associated protein 1A/1B-light chain 3 proteins (LC3)) and mitochondrial fission (through *Bcl-2-related ovarian killer protein* (BOK)), while inhibiting fusion (through mitofusin 1 and the optic atrophy protein (OPA1); see text for details. S1P generally activates autophagy, although the significance of this fact for mitochondrial turnover is not yet known. However, S1P augments mitochondrial assembly via protein kinase A (PKA), *peroxisome proliferator-activated receptor gamma coactivator 1* (PGC-1α), and prohibitin 2. S1P and ceramide regulate the anti-apoptotic Bcl-2 and the Bcl-2 family protein: Bcl_xL_, and pro-apoptotic Bax, Bad, or Bim. In turn, some of the proteins influence the enzymes of sphingolipid metabolism, ensuring negative or positive feedback regulation

Maintenance of mitochondrial dynamics is crucial for cell cell health. Non-symmetrical fission allows sequestration of damaged, dysfunctional, or “worn” mitochondrial material, which can be degraded in the process of mitophagy, while fusion allows amassing healthy organelles. Sphingolipid signaling has tight ties with mitochondrial dynamics. The levels of sphingoid bases which serve as sphingolipid precursors increases with yeast chronological aging, and these compounds inhibit mitochondrial fusion, leading to fragmentation and to age-related symptoms of mitochondrial decay (Fig. 4) [250]. Ceramide has been shown to activate fission of mitochondria in various tissues and in a cellular model, acting through modulation of expression levels of BOK (Bcl-2-related ovarian killer protein) [251]. Ceramide inhibits Akt signaling (synergistically with intracellular amyloid β_42_ (Aβ_42_)) and disturbs the fusion-fission regulation in neuronal cell lines through down-regulation of the fusion-promoting proteins mitofusin 1 and OPA1 (optic atrophy 1) [252] **(**Fig. 4**)**. Ceramide also reduced the levels of the fission regulator dynamin-related protein 1, although in muscle cells its seemed to exert opposite effect [252, 253]. Likewise, loss of the ceramide transfer protein CERT results in disturbed transport of ceramides from endoplasmic reticulum to Golgi apparatus, hexosylceramide accumulation in mitochondria, and lower frequency of both fusion and fission [254]. Finally, like in the plasma membrane, ceramide plays important structural roles in raft-like domains, and their disruption through inhibition of ceramide synthase disturbs the fission process [255].

Mitophagy, the mitochondria-targeting subset of autophagy, can be triggered by signs of organellar decay such as ROS generation or loss of mitochondrial membrane potential. The resulting autophagosome fuses with lysosome to create autophagolysosome, a process dependent on the LC3 (microtubule-associated protein 1A/1B-light chain 3) proteins. Mitophagy may result in either homoeostatic removal of damaged mitochondria, or escalate into various modes of cell death, depending on the circumstances. LC3 proteins interact with ceramide in mitochondrial membranes in an selective way dependent on the LC3 isoform, and this interaction facilitates autophagosome binding [256]. Besides other cellular sources, ceramide can be produced in mitochondria at least in some tissues; enzymes of its metabolism including CerS, sphingomyelinase, and ceramidase have been detected in isolated mitochondria [257–259]. Ceramide’s role in mitophagy can extend to mitophagic cell death [260].

Limited data suggests the involvement of SphKs in autophagy in general [180], suggesting possible links with mitochondrial quality assurance mechanisms. Moreover, S1P has been shown to activate mitochondrial biogenesis and adenosine triphosphate (ATP) generation via S1PR2, the protein kinase A (PKA)/cAMP response element-binding protein (CREB) pathway, and peroxisome proliferator-activated receptor γ coactivator 1α (PGC-1α) [261]. Mitochondrial assembly and the respiratory chain function are also dependent upon S1P binding to the prohibitin 2 protein [262]. Major part of S1P’s anti-apoptotic signaling occurs through its influence on mitochondria-associating proteins of the Bcl-2 family. S1P increases Bcl-2 level [263] and phosphorylation [264], leading to inhibition of apoptosis. S1P also down-regulates Bcl2-associated X protein (Bax), Bad, and Bim (BCL2-like 11 protein) [263, 265, 266]. In turn, Bcl-2 and Bcl_xL_ inhibit ceramide synthesis by nSMase [267]. Interestingly, Bcl-2 also increases SphK1 level and activity [268], and Bax/Bak activates CerS [269]. Although IIS is a classical regulator of survival, some of the mentioned effects are not mediated by PI3K/Akt signaling. SphK2-synthesized S1P promotes autophagy and the associated tissue tolerance to neurodegenerative ischemic insult (preconditioning in cortical neurons) [270], but it remains to be investigated how it would translate to any actual links with mitophagy.

#### Sphingolipids in the Alzheimer’s Disease

Accumulating evidence points to the involvement of sphingolipids in the neurodegeneration in AD. Hippocampal ceramide and sphingomyelin content correlate with age in men and aging in females leads to reduction in the fraction of phosphorylated sphingosine (S1P/sphingosine ratio), suggesting that age-related changes in bioactive sphingolipids might create pro-apoptotic, neurodegeneration-conductive environment [83]. Imbalance in the S1P and ceramide, which potentially might decide of the brain cell fates is observed from the earliest clinically recognizable stages of AD and correlates with Braak staging of neurodegenerative changes (Table 1) [76, 79]. The presence of a very early peak in ceramide generation in the brain has led to a proposal of a pre-*mild cognitive impairment* (MCI) stage of AD development [86]. The observed changes in sphingomyelin are less coherent [92]. However, using autopsy material from the Baltimore Longitudinal Study of Aging, Varma et al. have noted that three sphingomyelin species and one hydroxysphingomyelin associated with the progression along the prodromal and pre-clinical stages of AD. Moreover, higher levels of identified sphingomyelins and hydroxysphingomyelin associate with the risk of future conversion to AD [81]. The association of sphingolipid levels with either early AD stages, and the accessibility of body fluids for diagnostic purposes have led to suggestions of sphingolipids as potential AD biomarkers useful for early risk identification/diagnosis. Toledo et al. have found that some serum sphingomyelin species correlate with progression from MCI to full AD [82]. Likewise, the enzymes of sphingolipid metabolism are altered in AD in a manner correlating with disease progression. Examples of up-regulated genes include the ceramide-producing synthases *LASS1*, *LASS2* (coding for CerS1 and CerS2, respectively) and acid sphingomyelinase *ASM*; S1P lyase *SGPL1*, and serine palmitoyltransferase *SPTLC2* (which decides on the general ceramide/sphingolipid levels) were also increased, while the acid ceramidase *ASAH1*, C1P-generating *CERK*, or—less obviously—*LASS6* are reduced [76, 77]. We confirmed the trend towards reduced expression of S1P- and C1P-producing enzymes (*SPHKs*, *CERK*, S1P receptors) in human sporadic AD brains although there were slight differences in the types of genes/isoforms affected [271].

**Fig. 5:**
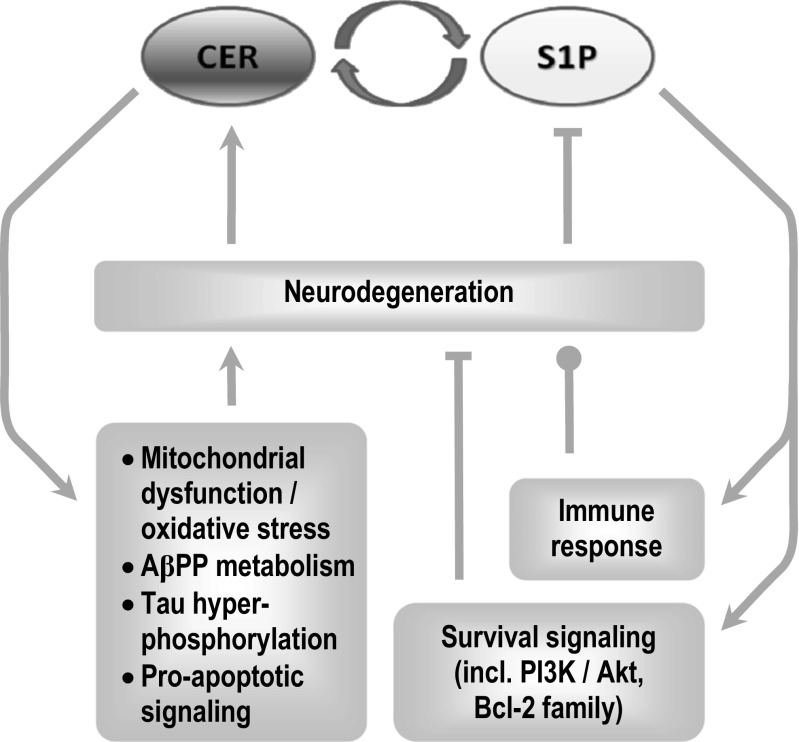
The significance of bioactive sphingolipids in neurodegeneration. The ‘sphingolipid rheostat’ model assumes antagonistic roles of ceramide and S1P in the regulation of cellular survival and death. Although exceptions have been identified, the tendency towards accumulation of ceramide and reduced levels of S1P still should generate strong neurodegenerative impulse. Potential downstream mechanisms include not only inhibition of survival signaling mostly mediated by the PI3K-Akt pathway, but also modulation of AβPP metabolism, and alteration of S1PR-dependent immune response—the latter capable of exerting either beneficial (Aβ clearance) or detrimental outcome (damage to neurons).  symbol denotes the ambiguous role of immune activation in neurodegenerative disorders (clearance of extracellular aggregates of misfolded proteins and debris from dying cells vs. creation of neurotoxic environment that accelerates the loss of neuronal connectivity and ultimately death of further neurons). S1P is known to modulate the immune response, but the possible outcome of the resulting reaction in the diseased brain is highly unclear

The roles of S1P and ceramide in the survival of brain neurons are far more complex than the antagonism described in the *sphingolipid rheostat* model. However, it is highly probable that changes in these compounds should significantly alter the rates of neuron degeneration and death **(**Fig. 5**)**. Modulation of IIS activity largely mediates the pro- or anti-apoptotic signaling of S1P, C1P, and ceramide. IGF-I resistance may be an important aspect of AD pathology, although over-activation of the pathway has also been suggested to contribute [272, 273]. Microglial expression of IGF-I reduces Aβ release and inflammation [274, 275], and IGF-I prevents Aβ_25–35_-induced hippocampal neuron death [276]. Human IGF-I-expressing cortex-derived neural stem cells have been proposed for AD therapy [277]. S1PR signaling can inhibit GSK-3β, the kinase engaged in tau phosphorylation, *via* PI3K-Akt [17]. S1P has been also shown to inhibit Aβ-dependent ceramide production by aSMase [278], although prolonged production of S1P by SPHK2 can lead to neurodegeneration [4, 40]. The roles of S1P in the regulation of secretion mechanisms also deserve more attention in the context of extracellular protein neurotoxicity [1, 279]. The pattern of sphingolipid metabolic enzyme changes in AD can be largely replicated in an animal Aβ precursor protein (AβPP, V717I)-transfected model, suggesting that these alterations arise in a relatively direct way in response to high Aβ peptide production [271]. Fingolimod effects on the age-dependent transcription of survival-regulating sphingolipid metabolism genes supports the need of its in-depth characterization as a potential disease-modifying treatment in AD and other neurodegenerative disorders [271].

## Concluding Remarks

Sphingolipids and sphingolipid metabolism are being increasingly implicated in aging and in age-related neurodegenerative disorders. The mechanisms of their engagement include both modulatory influences on membrane microenvironments (importantly, lipid rafts) as structural components, and interactions with signaling pathways. Crucially for aging and neurodegeneration, sphingolipids modulate neurotransmission and hormonal regulation. Sphingolipids’ cross-talk with the IGF-I-Akt-mTOR pathway may modulate multiple aspects of cellular survival, stress response, and aging. The potential significance of these interactions is vast and might include opportunities for therapeutic interventions. However, the depiction of sphingolipid engagement in long-term homoeostasis, requires much more comprehensive understanding. Currently available means of intervention involving sphingolipids need to be better understood and clinically refined before the compromise between their side-effects and the possible benefits becomes a viable option.
